# A self-reinforcing nanoplatform for triple-synergistic therapy: NIR-triggered photothermal/gas/chemodynamic therapy of tumors

**DOI:** 10.3389/fchem.2025.1742786

**Published:** 2025-12-08

**Authors:** Yan Xue, Xiaoxiao Chen, Xi Chen, Songhui Xue, Meijuan Qian, Dongzhi Wang

**Affiliations:** 1 Research Center of Molecular Medicine, Nantong Health Vocational College, Nantong, China; 2 Department of Hepatobiliary and Pancreatic Surgery, Affiliated Hospital of Nantong University, Nantong, China; 3 Key Laboratory of Neuroregeneration of Jiangsu and Ministry of Education, Co-innovation Center of Neuroregeneration, Nantong University, Nantong, China

**Keywords:** metal-organic framework, nitric oxide, chemodynamic therapy, photothermal therapy, gas therapy

## Abstract

**Introduction:**

A major challenge in nanomedicine is developing multifunctional nanoplatforms capable of achieving synergistic cancer therapy.

**Methods:**

In the present study, we developed a CD44-targeted nanocomposite, named UiO-SNO@CuS/HA, for efficacy evaluation in combination therapy including photothermal therapy (PTT), nitric oxide (NO) gas therapy and chemodynamic therapy (CDT) The nanoplatform was produced through the preparation of UiO-66-SH metal-organic framework (MOF) followed by the post-synthetic nitrosation of S-thiols to give S-nitrosothiols (SNO) as the NO donor. Afterward, in situ growth of ultrasmall CuS nanoparticles on the MOF surface led to the eventual coating of the hybrids with hyaluronic acid (HA) for active tumor targeting.

**Results:**

Under 1064 nm laser irradiation, the CuS component mediated effective PTT with a photothermal conversion efficiency of 41.4%. The generated photothermal heat also leads to the release of a considerable amount of the gas NO (135 μM, pH 4.6) and promotes the release the ions Cu^2^ + in the acidic tumor microenvironment. The Cu^2+^ that was released was reduced to Cu^+^ by glutathione, achieving GSH depletion of around 80%. This not only triggered a Fenton-like reaction with H_2_O_2_ to produce reactive hydroxyl radicals (·OH) for CDT, but also stimulated further production of NO from SNO moieties, forming a self-propagating therapeutic cycle. The series of events led to an increase of 4.2 times generation of intracellular reactive oxygen species (ROS), severe mitochondrial dysfunction with a decrease of 85% in membrane potential, and finally 78.4% apoptosis was induced in HeLa cells.

**Discussion:**

The triple-combination therapy generated by UiO-SNO@CuS/HA was demonstrated to have much higher cancer cell killing efficacy in vitro than either single or dual therapies, and very good biocompatibility with normal cells. This study reports a rationally designed feedback-amplified nanosystem that enables potent and specific triple-synergistic tumor therapy, representing a practical strategy for advanced combinatorial cancer therapy.

## 1 Introduction

Cancer remains one of the most devastating diseases affecting populations worldwide. According to the latest data from the International Agency for Research on Cancer, approximately 20 million new cancer cases and over 9.7 million cancer-related deaths occurred in 2022 (Bray et al., 2024). This escalating global health crisis underscores the urgent need for the development of more effective and safer treatment options. While conventional therapies such as surgery, chemotherapy, and radiotherapy have long been the cornerstone of cancer treatment, they are often associated with serious side effects, drug resistance, and a lack of precision—issues that contribute to suboptimal outcomes for many patients (Wang et al., 2016). This complex situation has spurred the rapid progression of innovative and targeted therapeutic approaches, with nanomaterial-based techniques emerging as particularly encouraging. Chemodynamic therapy (CDT) has emerged as a promising tumor-selective treatment that exploits Fenton or Fenton-like reactions to convert endogenous hydrogen peroxide (H_2_O_2_) into highly reactive hydroxyl radicals (·OH) (Lin et al., 2018; Qian et al., 2019; Tang et al., 2019). These radicals induce oxidative damage to essential biomolecules, ultimately leading to programmed cell death. The selectivity of CDT arises from the unique biochemical milieu of tumors, characterized by a mildly acidic microenvironment and elevated H_2_O_2_ levels, which minimizes off-target toxicity to surrounding healthy tissues (Chen et al., 2020; Liu et al., 2025; Ranji-Burachaloo et al., 2018; Zhang et al., 2018). Despite these advantages, the efficacy of CDT is often limited by intrinsic biological constraints. Elevated intracellular glutathione (GSH) concentrations in cancer cells effectively scavenge ·OH, reducing oxidative stress and compromising therapeutic potency (Tian et al., 2021). Furthermore, insufficient H_2_O_2_ availability and the suboptimal catalytic activity of conventional metal ions (e.g., Fe^2+^) under near-neutral tumor pH further restrict Fenton efficiency. To address these challenges, extensive efforts have focused on engineering nanoplatforms capable of depleting GSH, enhancing endogenous H_2_O_2_ generation, and integrating CDT with complementary therapeutic modalities for synergistic effects (Wang et al., 2020).

Recently, cancer therapies that utilize gas molecules have attracted considerable interest as a powerful complement to traditional treatment methods (He, 2017; Yu et al., 2018). Various gasotransmitters including nitric oxide (NO) (Wu et al., 2020; Xiang et al., 2024), carbon monoxide (CO) (Ning et al., 2024; Wang et al., 2024; Wang et al., 2025a), hydrogen sulfide (H_2_S) (Rong et al., 2023), sulfur dioxide (SO_2_) (He et al., 2024; Wang et al., 2025b) have been thoroughly investigated for their roles in inhibiting cell growth, enhancing tumor sensitivity to standard therapies, and triggering apoptosis. Of the naturally occurring gaseous messengers, NO is particularly notable for its diverse biological functions. At higher concentrations, NO can induce nitrosative stress, impair mitochondrial operations, and activate apoptotic pathways, resulting in significant cytotoxic effects on cancer cells (Kim et al., 2014). However, the pathway to clinical application of NO-based treatments is not without obstacles, mainly due to the challenges associated with achieving accurate, localized, and controllable delivery. Early release of NO could result in systemic adverse effects, while inadequate accumulation at tumor locations diminishes therapeutic potential (Jiang et al., 2024). Consequently, the strategic development of stimuli-responsive nanoplatforms capable of releasing NO and allowing for spatiotemporal control has become a key area of focus in the progress of gas-assisted cancer therapies.

**SCHEME 1:**
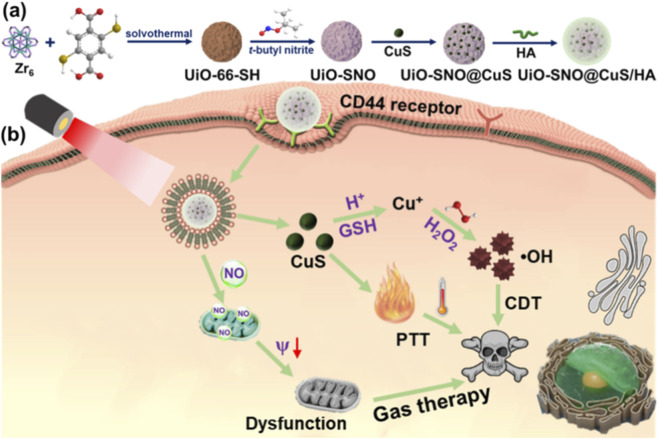
Schematic illustration of UiO-SNO@CuS/HA synthestic process **(a)** and application for synergistic gas/PTT/CDT therapy **(b)**.

It is important to note that advances in therapeutic strategies are closely accompanied by progress in diagnostic technologies. Emerging nanoscale imaging methods and highly sensitive electrochemical biosensors are being developed to precisely track tumor progression and treatment responses, offering powerful tools for early detection and improved patient prognosis (Marcuello et al., 2025; Raj et al., 2025). The integration of these accurate monitoring techniques with targeted and efficient therapies, such as gas therapy, is essential for enhancing comprehensive cancer management. This synergy further drives the development of sophisticated “all-in-one” nanoplatforms capable of uniting diagnosis and treatment within a single system. Herein, we have rationally designed a self-reinforcing nanoplatform, UiO-SNO@CuS/HA, which integrates a triple-synergistic therapy (PTT, NO gas therapy, and CDT) into a single material for effective tumor suppression. As illustrated in Scheme 1, UiO-66-SH metal–organic framework (MOF) nanoparticles were synthesized using Zr^4+^ and 2,5-dimercapto-1,4-benzenedicarboxylic acid (H_2_DMBD) as building blocks. The surface thiol groups were subsequently converted to S-nitrosothiol (SNO) moieties to form a thermally responsive NO donor (UiO-SNO). Ultrasmall CuS nanoparticles (NPs) were then grown *in situ* on the MOF surface and the composite was further coated with hyaluronic acid (HA) to endow CD44-targeting capability (Scheme 1a). Upon accumulation in tumor tissue and irradiation with a 1,064 nm near infrared-II (NIR-II) laser, CuS generates localized photothermal heating, which simultaneously accelerates NO release and enhances Cu^2+^ liberation. Then, the released Cu^2+^ is reduced to Cu^+^ by intracellular GSH and initiated a cascade that involves GSH depletion, Cu^+^-driven Fenton-like reaction for ·OH generation. This self-amplifying feedback loop intensifies oxidative and nitrosative stress, ultimately leading to mitochondrial collapse and apoptosis (Scheme 1b). Collectively, these findings underscore a rationally engineered, feedback-amplified nanosystem that integrates targeted delivery with cascade-enhanced multimodal therapy, providing a versatile framework for next-generation tumor theranostics.

## 2 Experiment section

### 2.1 Synthesis of UiO-66-SH, UiO-SNO, UiO-SNO@CuS/HA

#### 2.1.1 Preparation of UiO-66-SH NPs

UiO-66-SH was synthesized according to the previous study (Hu et al., 2022). Typically, ZrCl_4_ (41.2 mmol) and H_2_DMBD (41.3 mmol) were dissolved in 40 mL of DMF solution by sonication for 30 min. Then, 750 μL of acetic acid was added in the above mixture and stirred for 10 min. The mixture was transferred to a Teflon-lined autoclave and heated at 120 °C for 12 h. The obtained products were centrifuged and wash with DMF and ethanol. At last, the products were dried under vacuum for 24 h at 60 °C. The typical yield of this synthesis, calculated based on the mass of the obtained UiO-66-SH product relative to the initial mass of ZrCl_4_, was in the range of 75%–80%.

#### 2.1.2 Preparation of UiO-SNO NPs

To introduce SNO functional groups, UiO-66-SH (10.0 mg) was dispersed in a methanol/toluene mixture (v/v = 1:9) containing excess t-butyl nitrite and stirred at 25 °C for 12 h. The product was rinsed with ethanol and deionized water and subsequently dried under vacuum for storage in the dark. The nitrosation reaction proceeded efficiently with a conversion rate exceeding 90%, as confirmed by UV-Vis spectroscopy. The mass yield of the isolated UiO-SNO product was approximately 85%.

#### 2.1.3 Preparation of UiO-SNO@CuS/HA NPs

For *in situ* CuS growth, 3.0 mL of 4 mM CuCl_2_ solution was mixed with a 1.0 mg/mL UiO-SNO suspension and stirred at 25 °C for 2 h. Subsequently, 240 μL of freshly prepared 50 mM Na_2_S solution was added, followed by heating at 90 °C for 10 min. The product (UiO-SNO@CuS) was collected via centrifugation, thoroughly washed, and lyophilized in the dark. To introduce the HA coating, UiO-SNO@CuS (2.0 mg) was dispersed in 10.0 mL of 1 mg/mL sodium hyaluronate solution and stirred overnight. The final product (UiO-SNO@CuS/HA) was recovered by centrifugation and freeze-drying.

### 2.2 Photothermal conversion experiments

The photothermal performance of UiO-SNO@CuS/HA NPs was systematically evaluated. Aqueous dispersions at various concentrations (0, 50, 100, 150, and 200 μg/mL) were exposed to a 1,064 nm NIR laser (1.0 W/cm^2^) for 10 min, with temperature recorded every 30 s using an infrared thermal imager. Deionized water served as the control. To determine the photothermal conversion efficiency (η), a 200 μg/mL dispersion was irradiated for 14 min and then allowed to cool naturally; the cooling phase data were used for calculation, with the entire process monitored by thermal imaging. Furthermore, the photothermal stability was assessed by subjecting a 100 μg/mL dispersion to five consecutive laser on/off cycles (10 min irradiation/cycle), demonstrating consistent temperature elevation upon repeated exposure.

### 2.3 NO generation

NO release quantification was done using the Griess assay, which detects nitrite (NO_2_^−^) as a stable oxidative product of NO. The absorbance value at 543 nm was measured by reacting different concentrations of NaNO_2_ (10–100 μM) with the Griess reagent under acidic conditions to construct a standard curve. For the NO release study, UiO-66-SNO or UiO-SNO@CuS/HA NPs (2.5 mg) were dispersed in 5 mL of PBS at different pH values (4.6, 6.0, 7.4) and incubated at 37 °C in the dark. Aliquots of 1 mL were taken every 10 min and centrifuged. The supernatant was reacted with the Griess reagent for absorbance measurement whereas the nanoparticles were re-dispersed in fresh PBS for the further release monitoring. To investigate the photothermal effect under NIR laser irradiation (1.0 W/cm^2^), a concomitant experiment was performed at pH 4.6 and 1,064 nm laser. All measurements were carried out in triplicate, with freshly prepared solutions, and absorbances were recorded within 10 min of the addition of Griess reagent.

### 2.4 Release of Cu^+^

The Fenton-like reaction between Cu^+^ and H_2_O_2_ produces ·OH that measured by a DPBF. The release of Cu^+^ ions from the NPs was estimated using this reaction. For the release study, UiO-SNO@CuS/HA NPs (2.0 mg/mL) were incubated in PBS containing GSH (200 μg/mL) at pH 4.6 and 7.4 at 37 °C with stirring. Aliquots of 1.0 mL were collected at predetermined time intervals including every 10 min for the first hour and subsequently every hour. These were centrifuged. The supernatant was treated with H_2_O_2_ and DPBF to analyse. The standard curve was used to generate cumulative Cu^+^ release profiles at both pH levels.

### 2.5 Measurement of mitochondrial membrane potential

HeLa cells were seeded in 6-well plate, and after 24 h incubation, treated with PBS, UiO-SNO, UiO-66-SH@CuS, and UiO-SNO@CuS/HA, after 4 h incubated, groups of 3 were irradiated upon 1,064 nm laser (1.0 W/cm^2^) for 10 min, and then incubated for another 4 h. The cells were then subjected to a 20-min staining process with JC-1 (10 μM). After that, JC-1 solution was removed, and washed three times with PBS. The red fluorescence (from J-aggregates) and green fluorescence (from monomers) of JC-1 were detected and captured using fluorescence microscopy. The specific excitation/emission wavelengths were set at 585/590 nm for the red fluorescence (J-aggregates) and 514/529 nm for the green fluorescence (monomers).

### 2.6 *In Vitro* cytotoxicity

The standard MTT assay is employed to assess the cytotoxicity of nanomaterials on HeLa cells. Briefly, HeLa cells were seeded in a 96-well plate at a density of 1 × 10^4^ cells per well in 200 µL of medium and allowed to adhere overnight in a 37 °C incubator with 5% CO_2_. Subsequently, the cells were treated with varying concentrations of UiO-SNO or UiO-SNO@CuS/HA and incubated for 48 h. To evaluate the photothermal effect, several wells were irradiated with a 1,064 nm laser (1.0 W/cm^2^ for 10 min) following incubation, and then cultured for an additional 12 h. A control group that received only laser treatment (without NPs) was also included in the experiment. After this, 10 µL of MTT solution (5.0 mg/mL) was added to each well and incubated for 4 h. Subsequently, 150 µL of DMSO was added to dissolve the formazan crystals, and the absorbance of the resulting solution was measured at 490 nm using a microplate reader.

### 2.7 Imaging of NO in living cells

The fluorescent probe DAF-FM DA could visualize the intracellular generation of NO. HeLa cells were treated with either UiO-SNO, UiO-SNO@CuS/HA, or UiO-SNO@CuS/HA plus 1,064 nm laser irradiation (1.0 W/cm^2^). Subsequent to the respective treatment, the cells were washed with PBS and co-stained with DAF-FM DA and Hoechst 33342, for labelling NO and nuclei, respectively. After that, confocal laser scanning microscopy (CLSM) observed the fluorescence.

### 2.8 Live/dead staining experiment

Live/dead cell viability was assessed using a Calcein-AM/PI double staining kit. HeLa cells were cultured in six well plates at 3 × 10^5^ cells per well for 24 h. The cells were treated with different nanomaterials for 6 h: UiO-SNO, UiO-SNO@CuS, UiO-66-SH@CuS, or UiO-SNO@CuS/HA. In the appropriate cases, designated groups received 1,064 nm laser irradiation for 10 min at 1.0 W/cm^2^. Following another 18 h incubation, cells were thoroughly washed with PBS and stained for 30 min at 37 °C with working solution containing Calcein-AM (2 µM) and PI (4 µM). Finally, they washed the cells with PBS to eliminate the excess dye and photographed them directly using CLSM to separate the live (green) cells and dead (red) cells.

## 3 Results and discussion

### 3.1 Synthesis and characterization of UiO-SNO@CuS/HA NPs

**FIGURE 1:**
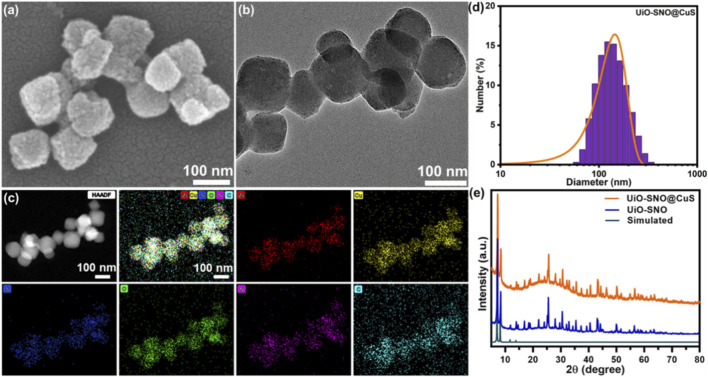
UiO-SNO@CuS/HA NPs: **(a)** SEM image; **(b)** TEM image; **(c)** element mapping images; **(d)** DLS data; **(e)** XRD pattern.

The successful construction of the multifunctional nanoplatform, UiO-SNO@CuS/HA, was systematically confirmed by a series of morphological and structural characterizations. First, the morphology of the as-synthesized UiO-66-SH MOF was examined. As revealed by scanning electron microscopy (SEM) and transmission electron microscopy (TEM) images (Supplementary Figure S1), the pristine UiO-66-SH nanoparticles exhibited a monodisperse polyhedral structure with a smooth surface. This well-defined starting morphology provides a critical baseline for tracking subsequent modifications. Following the *in situ* growth of CuS, the surface of the nanoparticles became distinctly rough and was decorated with numerous ultrasmall nanoparticles (Figures 1a,b). This marked morphological change provided direct evidence for the successful formation of the UiO-SNO@CuS core-shell structure. Moreover, the surface topography was quantitatively analyzed by atomic force microscopy (AFM). The calculated root-mean-square roughness (Rq) value significantly increased from 2.1 nm for UiO-66-SH to 18.7 nm for UiO-SNO@CuS/HA (Figure 1c), providing further compelling evidence for the successful material synthesis through this pronounced change in surface morphology. As shown in Figure 1c, the results of elemental mapping revealed that the uniform spatial distribution of Zr, O, S, and Cu across the composite, further confirming the successful coating of CuS NPs into MOF surface. The loading capacity of CuS was measured to be 9.8 wt% by ICP-OES. The obtained composite of UiO-SNO@CuS/HA have an average diameter with 155.3 ± 6.2 nm with a polydispersity index (PDI) of 0.15, confirming its suitable size and narrow distribution for enhanced cellular uptake (Figure 1d). In Figure 1e, XRD patterns indicated that the crystalline structure of UiO-66-SH was maintained after the nitrosation and CuS NPs deposition processes (Hu et al., 2022). Collectively, these findings confirm that UiO-SNO@CuS/HA retains its ordered architecture and exhibits physicochemical characteristics that are favorable for therapeutic applications.

**FIGURE 2:**
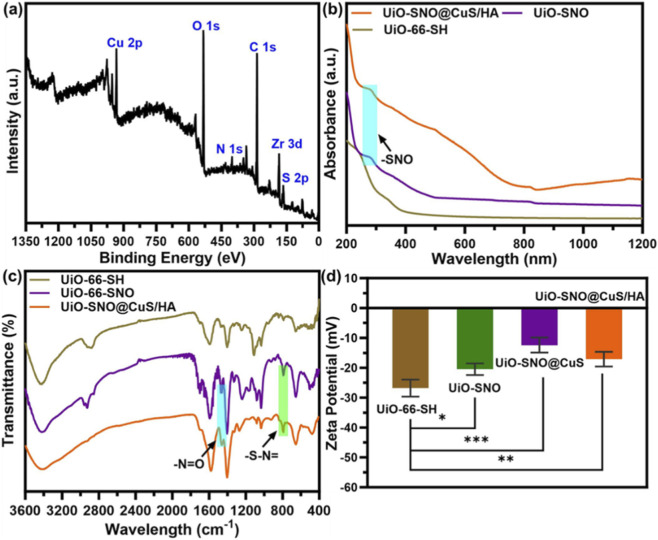
**(a)** XPS spectrum of UiO-SNO@CuS/HA NPs. **(b)** UV-vis spectrum; **(c)** element mapping images; **(c)** FTIR spectra and **(d)** Zeta potential of UiO-66-SH, UiO-SNO, UiO-SNO@CuS, and UiO-SNO@CuS/HA.

The XPS survey spectrum (Figure 2a) confirmed the presence of Zr, C, O, S, and Cu, consistent with the elemental distribution observed by EDS. UV-Vis spectra (Figure 2b) revealed a new absorption peak around 300 nm for both UiO-SNO and UiO-SNO@CuS/HA, corresponding to the SNO groups, which confirmed the successful nitrosation (Yin et al., 2020). FTIR spectra (Figure 2c) showed characteristic absorption bands at 1,602 cm^−1^ and 787 cm^−1^, corresponding to the N=O and S-N bonds, respectively, further verifying the presence of NO donors in the composite (Wang et al., 2021; Zhang et al., 2020; Zhao et al., 2020). Zeta potential measurements (Figure 2d) showed stepwise changes in surface charge after each modification, which supports the successful assembly of UiO-SNO@CuS/HA.

### 3.2 Photothermal conversion of UiO-SNO@CuS/HA

**FIGURE 3:**
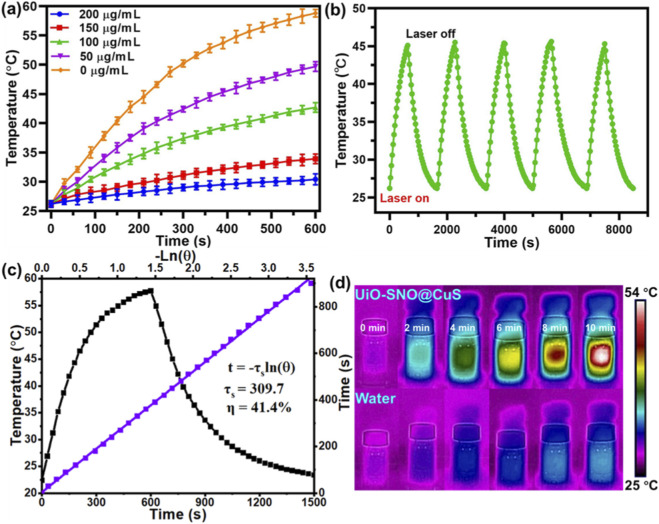
**(a)** The temperature elevation of the aqueous dispersion of UiO-SNO@CuS/HA with different concentration. **(b)** Temperature curves of UiO-SNO@CuS/HA with concentrations of 100 μg/mL for five laser on/off cycles. **(c)** Calculation of the photothermal-conversion efficiency value of UiO-SNO@CuS/HA at 1,064 nm. **(d)** *In vitro* infrared thermal imaging of UiO-SNO@CuS/HA and water solution under the 1,064 nm laser irradiation at the specific time with power density of 1.0 W/cm^2^.

The photothermal performance of the UiO-SNO@CuS/HA nanocomposite was evaluated under NIR laser irradiation (1,064 nm, 1.0 W/cm^2^). As shown in Figure 3a, dispersions of UiO-SNO@CuS/HA exhibited a concentration-dependent temperature increase after 10 min of laser exposure, with a marked rise in temperature observed at higher concentrations. At 200 μg/mL, the temperature increased by 31.6 °C, confirming a strong photothermal effect. For photothermal stability, the nanocomposite was subjected to five consecutive on/off cycles of laser irradiation (Figure 3b), demonstrating consistent temperature elevation across all cycles, thus proving its excellent photostability. The photothermal conversion efficiency (η) was calculated using the cooling curve of the 200 μg/mL dispersion after 14 min of irradiation (Figure 3c), yielding an efficiency of 41.4%. This high photothermal conversion efficiency indicates that UiO-SNO@CuS/HA is a highly effective photothermal agent. Infrared thermal imaging (Figure 3d) visually confirmed the progressive temperature rise in the nanocomposite dispersion under NIR irradiation, contrasting sharply with the negligible temperature increase observed for the control (water). These results demonstrate that UiO-SNO@CuS/HA is an efficient and stable photothermal agent for tumor therapy.

### 3.3 pH-triggered Cu^2+^ release and photothermal-enhanced NO gas release

**FIGURE 4:**
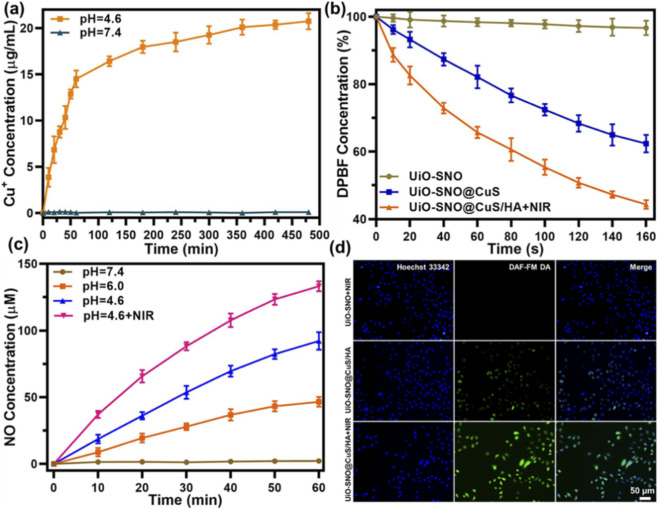
**(a)** Cu^+^ release profiles of UiO-SNO@CuS/HA in different pH solutions with 2.0 mM GSH. **(b)** Time-dependent DPBF degradation curves under different conditions. **(c)** NO release profiles of UiO-SNO@CuS/HA in different PBS solutions. **(d)** Fluorescence images of HeLa cells after incubation UiO-SNO@CuS/HA in the presence/absence of 1,064 nm laser. The generation of NO was detected by DAF-FM DA.

The release profiles of Cu^2+^ and NO were examined under various conditions to evaluate the responsiveness of UiO-SNO@CuS/HA to tumor-specific stimuli. Figure 4a illustrates the pH-dependent release of Cu^+^ ions. At neutral pH (7.4), minimal Cu^+^ release was observed. In contrast, at acidic pH (4.6), Cu^+^ release was significantly enhanced, reaching approximately 20 μg/mL within 80 min. This pH-triggered release is essential for minimizing toxicity to healthy cells, as the acidic environment of the tumor facilitates the controlled liberation of Cu^+^ ions, which are critical for the Fenton-like reactions that drive CDT. Subsequently, the generation of ·OH was quantitatively assessed using DPBF as a fluorescent probe. As illustrated in Figure 4b, the UiO-SNO@CuS significantly degraded DPBF in the presence of H_2_O_2_, likely facilitated by ·OH produced through the Cu^+^-mediated Fenton-like reaction. This degradation was markedly enhanced by NIR laser irradiation. The increased decay of DPBF under photothermal conditions suggests that localized heating promotes a greater release of Cu^2+^, which is further reduced to Cu^+^, thereby enhancing ·OH generation and reinforcing the self-cycling therapeutic process.

The NO gas release from the UiO-SNO@CuS/HA nanocomposites was performed by a typical Griess assay under different pH solutions. As shown in Figure 4c, the amount of NO gas released is negligible in pH 7.4 solution. Subsequently, the amount of NO released gradually increases as the pH of the solution decreases. For example, the release amount reaches 52 μM at a pH of 6.0 and increases to 105 μM at a pH of 4.6. Notably, the release amount rapidly escalates to 135 μM when exposed to NIR light irradiation. This phenomenon is attributed to the release of Cu^+^, which is triggered by the acidic environment and the photothermal effect, both of which synergistically enhance the release of NO gas. The combination of these two factors greatly enhanced the NO release, providing a robust means to induce apoptosis in tumor cells. Fluorescence imaging using DAF-FM DA (Figure 4d) confirmed the efficient NO generation in HeLa cells, further highlighting the efficacy of the UiO-SNO@CuS/HA platform in facilitating targeted NO release.

### 3.4 ROS generation and mitochondrial dysfunction

To validate the proposed self-reinforcing mechanism, we first investigated the depletion of intracellular GSH, a central process in our therapeutic cycle. The GSH levels in HeLa cells after various treatments were quantified using the DTNB assay (Supplementary Figure S2). Consistent with our hypothesis, the UiO-SNO@CuS/HA nanocomposite induced the most pronounced GSH depletion, with the maximum reduction achieved after 4 h of incubation. This can be attributed to the efficient reduction of the released Cu2^+^ to the active Cu^+^ state, which concurrently consumes GSH. This critical depletion has a dual effect: it directly lowers the cellular antioxidant defense capability, and it facilitates the continuous generation of Cu^+^ to sustain the therapeutic cascade. The resultant pro-oxidant intracellular environment sets the stage for the massive ROS burst observed in the subsequent experiments.

**FIGURE 5:**
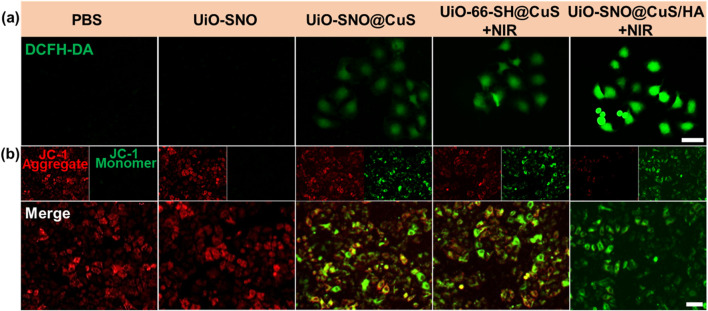
**(a)** Fluorescence images of cellular ROS against HeLa cells after various treatments, detected via the DCFH-DA probe. **(b)** Evaluation of intracellular MMP in HeLa cells using JC-1 staining under diverse treatment conditions. Scale bar = 50 μm.

The intracellular generation of ROS was measured using the DCFH-DA probe. Figure 5a shows that cells treated with UiO-SNO@CuS/HA under NIR irradiation exhibited significantly higher ROS production compared to the control groups, confirming that the synergistic effects of PTT, CDT, and NO gas therapy lead to a substantial ROS burst. The presence of Cu^+^ ions from CuS and NO release from the SNO moieties catalyzed Fenton-like reactions, generating highly toxic ·OH, which contributed to oxidative stress.

Given that excessive ROS and elevated levels of NO have been reported to impair mitochondrial function, we subsequently assessed alterations in mitochondrial membrane potential (MMP) using the JC-1 fluorescent probe assay. As illustrated in Figure 5b, cells treated with UiO-SNO@CuS/HA and subjected to NIR irradiation displayed a total loss of MMP, evidenced by intense green fluorescence emission. The MMP depolarization serves as a critical hallmark of mitochondrial dysfunction, ultimately triggering apoptotic cell death. These findings indicate that the synergy between ROS production and NO-mediated mitochondrial impairment contributes to the improved antitumor activity of the nanoplatform.

### 3.5 *In vitro* therapeutic efficacy of UiO-SNO@CuS/HA nanoplatform

**FIGURE 6:**
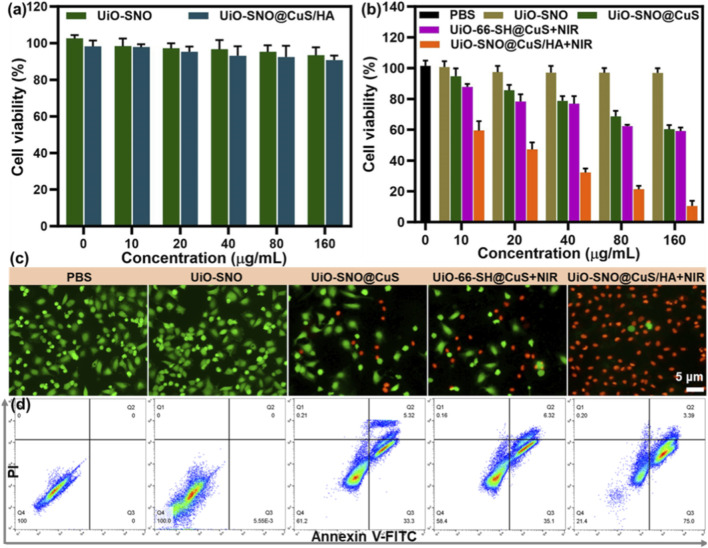
**(a)** Viabilities of HL-7702 treated with UiO-SNO and UiO-SNO@CuS/HA. **(b)** Cytotoxicity in HeLa cells after treatment with different formulations. n = 6. **(c)** Live/dead staining with Calcein-AM/PI. **(d)** Flow cytometric analysis of therapeutic effects on HeLa cells through different treatments.

Given its considerable potential as an antitumor therapeutic agent, we conducted a systematic investigation into the antitumor performance and biocompatibility of the UiO-SNO@CuS/HA nanocomposite. First, the 3-(4,5-dimethylthiazol-2-yl)-2,5-diphenyltetrazolium bromide (MTT) assay was employed to evaluate cytotoxicity. The cytotoxicity of the UiO-SNO@CuS/HA nanoplatform was assessed in both normal human hepatocyte HL-7702 cells and HeLa cancer cells. Figure 6a shows that UiO-SNO@CuS/HA exhibited excellent biocompatibility with HL-7702 cells, maintaining cell viability above 90% even at high concentrations (160 μg/mL). This suggests that the nanoplatform poses minimal toxicity to normal cells, a crucial consideration for potential clinical applications. In comparison, UiO-SNO@CuS and UiO-66-SH@CuS + NIR treatment at the same concentration caused 52% and 54% cell death respectively (Figure 6b). Conversely, when HeLa cells treated with UiO-SNO@CuS/HA under NIR irradiation, the cell viability was decreased to 10.5%, which can be attributed to the synergistic effects of PTT, gas therapy, and CDT.

In order to visually confirm the results of the cell viability assay, a co-staining experiment was conducted using the Calcein-AM/I double staining method to validate the MTT results. In Figure 6c, nearly all HeLa cells treated with UiO-SNO@CuS/HA and subsequently exposed to 1,064 nm laser irradiation exhibited red fluorescence, indicating significant cytotoxicity under these conditions. Additionally, flow cytometry provided additional confirmation of these findings (Figure 6d). The synergistic therapy was confirmed to induce significant apoptosis, with apoptotic rates of 38.6% and 41.4% for UiO-SNO@CuS and UiO-66-SH@CuS + NIR, respectively. However, the combination of all therapeutic modalities in UiO-SNO@CuS/HA + NIR resulted in a dramatically enhanced apoptotic rate of 78.4%. The aforementioned findings align with the results obtained from the MTT and live/dead staining assays. It can be concluded that upon NIR activation, the UiO-SNO@CuS/HA nanoplatform can synergistically induce apoptosis in cancer cells, primarily due to PTT, NO therapy, and CDT, while maintaining good biocompatibility with normal cells.

## 4 Conclusion

In this study, we successfully developed a CD44-targeted nanoplatform (UiO-SNO@CuS/HA) that integrates three synergistic therapeutic modalities—PTT, NO gas therapy, and CDT—into a single multifunctional system. The unique design of this nanoplatform allows for an internally amplified feedback cycle in the TME, where (1) CuS nanoparticles generate localized heat upon NIR irradiation to induce PTT; (2) heat triggers the release of Cu^2+^ and NO; and (3) Cu^+^ ions catalyze Fenton-like reactions to generate highly cytotoxic ·OH, while enhancing NO release. This self-reinforcing feedback loop significantly enhances the overall therapeutic efficacy by inducing substantial oxidative stress and mitochondrial dysfunction, which in turn triggers apoptosis in malignant cells. *In vitro* evaluations demonstrate that this triple-modality treatment achieves significantly superior tumor cell killing compared to any single or dual-modality therapy, while exhibiting excellent biocompatibility with normal cells. Looking forward, this work not only establishes a conceptual foundation for synergistic oncotherapy but also outlines clear pathways for future development. Subsequent research will focus on systematic *in vivo* assessments of biodistribution and therapeutic efficacy, deeper exploration of the molecular-level mechanisms, and the integration of imaging functionalities to develop a truly versatile theranostic platform. This multifunctional nanoplatform, therefore, presents a robust and promising strategy for advanced combinatorial cancer treatment.

## Data Availability

The original contributions presented in the study are included in the article/Supplementary Material, further inquiries can be directed to the corresponding authors.
