# Supplementary material for: A self-reinforcing nanoplatform for triple-synergistic therapy: NIR-triggered photothermal/gas/chemodynamic therapy of tumors
Source: Front Chem. 2025 Dec 8;13:1742786. doi: 10.3389/fchem.2025.1742786 (PMC12722887; doi:10.3389/fchem.2025.1742786)
Supplement: Supplementary file 1 [file DataSheet1.pdf]

Supporting Information

A Self-Reinforcing Nanoplatform for Triple-Synergistic Therapy: NIR-Triggered Photothermal/Gas/Chemodynamic Therapy of Tumors

Yan Xue[1, #], Xiaoxiao Chen[2, #], Xi Chen[3, #], Songhui Xue[1], Meijuan Qian[1, *], Dongzhi Wang[2, *]

[1]Research Center of Molecular Medicine, Nantong Health Vocational College, Nantong 226010, China.

[2]Department of Hepatobiliary and Pancreatic Surgery, Affiliated Hospital of Nantong University, Nantong 226006, China.

[3] Key Laboratory of Neuroregeneration of Jiangsu and Ministry of Education, Co-innovation Center of Neuroregeneration, Nantong University, Nantong, 226007, China.

# These three authors contributed equally to this study.

*** Correspondence:**
Meijuan Qian, Prof. Email: 158142474@qq.com；
Dongzhi Wang, Ph.D. Email: w_dongzhi@163.com.

1. **Figures**

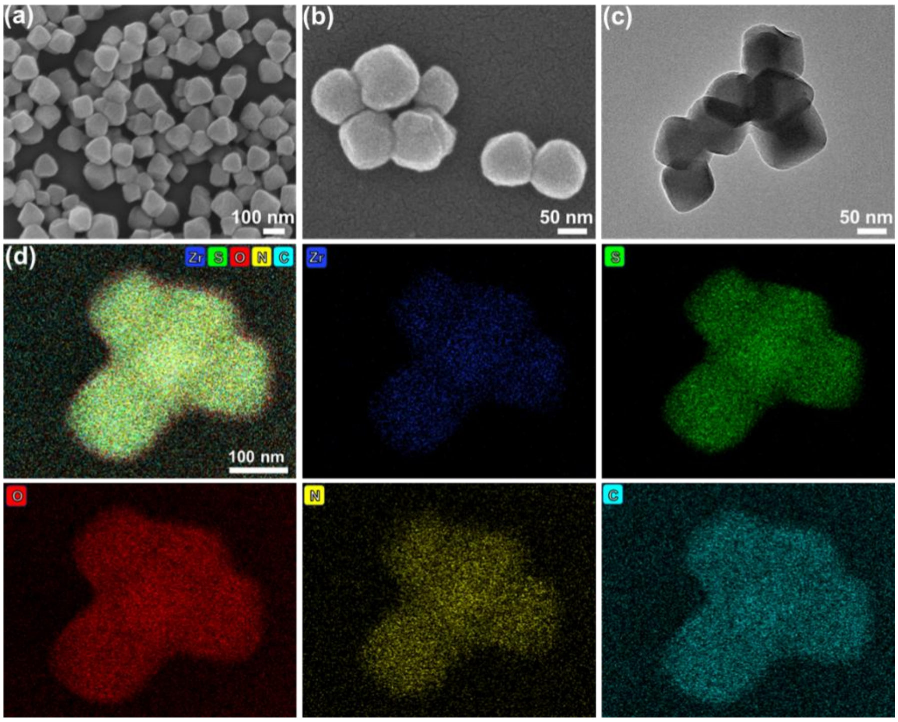

Figure S1. (a) and (b) SEM images of UiO-66-SH nanoparticles; (c) TEM image; (d) element mapping images.

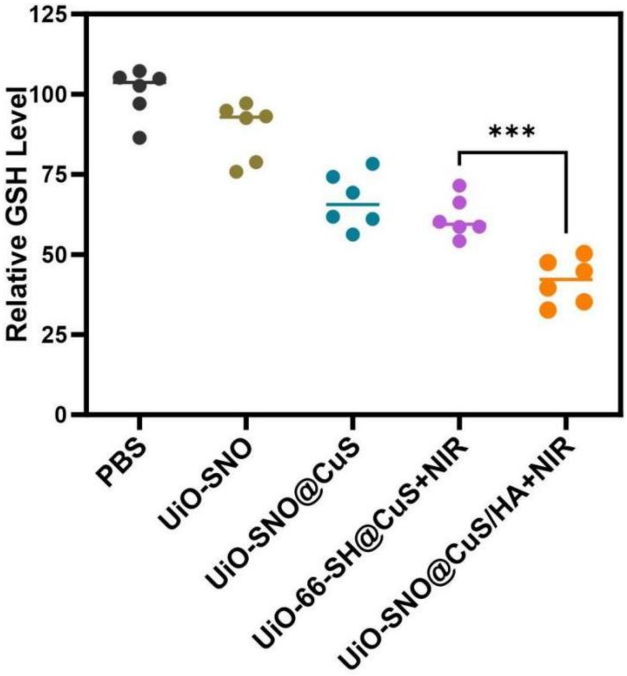

Figure S2. Relative GSH level of HeLa cells after treatment with PBS, UiO-SNO, UiO-SNO@CuS, UiO-66-SH@CuS+NIR, and UiO-SNO@CuS/HA+NIR.
